# Catecholaminergic Polymorphic Ventricular Tachycardia and Gene Therapy: A Comprehensive Review of the Literature

**DOI:** 10.7759/cureus.47974

**Published:** 2023-10-30

**Authors:** Elvis Henriquez, Edwin A Hernandez, Sravya R Mundla, Diptish H Wankhade, Muhammad Saad, Sagar S Ketha, Yasaswini Penke, Gabriela C Martinez, Faiza S Ahmed, Muhammad Sheheryar Hussain

**Affiliations:** 1 Miscellaneous, Facultad de Medicina, Universidad de Ciencias Medicas, Las Tunas, CUB; 2 Miscellaneous, Faculty of Medicine, Universidad de El Salvador, San Salvador, SLV; 3 Internal Medicine, Apollo Institute of Medical Sciences and Research, Hyderabad, IND; 4 Internal Medicine, Terna Medical College, Navi Mumbai, IND; 5 Internal Medicine, Fatima Memorial College (FMH) of Medicine and Dentistry, Lahore, PAK; 6 Internal Medicine, Government Medical College, Srikakulam, IND; 7 Internal Medicine, Faculty of Medicine, Universidad Nacional Autonoma de Honduras, San Pedro Sula, HND; 8 Internal Medicine, Advocate Lutheran General Hospital, Park Ridge, USA; 9 Internal Medicine, Dow University of Health Sciences, Civil Hospital Karachi, Karachi, PAK

**Keywords:** gene therapy, tachycardia, arrhythmia, channelopathy, catecholaminergic polymorphic ventricular tachycardia

## Abstract

Catecholaminergic polymorphic ventricular tachycardia (CPVT) is an inherited channelopathy. In this review, we summarize the epidemiology, pathophysiology, clinical characteristics, diagnostics, genetic mutations, standard treatment, and the emergence of potential gene therapy. This inherited cardiac arrhythmia presents in a bimodal distribution with no association between sex or ethnicity. Six different CPVT genes have been identified, however, most of the cases are related to a heterozygous, gain-of-function mutation on the ryanodine receptor-2 gene (RyR2) and calsequestrin-2 gene (CASQ2) that causes delayed after-depolarization. The diagnosis is clinically based, seen in patients presenting with syncope after exercise or stress-related emotions, as well as cardiac arrest with full recovery or even sudden cardiac death. Standard treatment relies on beta-blockers, with add-on therapy, flecainide, and cardiac sympathetic denervation as second-line treatments. An implantable cardioverter-defibrillator is indicated for patients who have suffered a cardiac arrest. Potential gene therapy has emerged in the last 20 years and accelerated because of associated viral vector application in increasing the efficiency of prolonged cardiac gene expression. Nevertheless, human trials for gene therapy for CPVT have been limited as the population is rare, and an excessive amount of funding is required.

## Introduction and background

Inherited arrhythmia syndromes encompass a wide array of disorders that present with symptoms from asymptomatic to sudden cardiac death [1]. The recognized patterns of inherited arrhythmias include long QT syndrome (LQTS), short QT syndrome, Brugada syndrome, catecholaminergic ventricular tachycardia, early repolarization syndrome, and idiopathic ventricular fibrillation. A meaningful discussion of all these syndromes is beyond the scope of this review; this article examines the potential of gene therapy for patients with catecholaminergic polymorphic ventricular tachycardia (CPVT).

CPVT is a highly malignant, inherited, potentially fatal ventricular arrhythmia triggered by exercise or high emotion, i.e., adrenergically mediated. It is classified as channelopathy, indicating a gene mutation that translates to specific ion channels in the cardiac myocytes, namely ryanodine receptor-2 (RyR2) and calsequestrin-2 (CASQ2) channels [1]. These altered channels disrupt the balance of ion currents responsible for the automaticity of electrical activity within the heart and predispose the heart to life-threatening arrhythmia production. It is a rare condition when compared to other channelopathies such as LQTS and Brugada syndrome; however, it may be more lethal as it presents without warning, and its most common symptom, syncope, may be misclassified and misattributed to other etiologies. Its incidence is widely quoted as 1:10,000 [2], but this is likely an underestimate of the true incidence due to the high clinical suspicion required for diagnosis. It is characterized by an increased incidence of fatal and near-fatal cardiac events [3]. These individuals have normal baseline electrocardiogram (ECG or EKG) and an anatomically normal heart and require genetic testing or classic clinical features for diagnosis. This is also complicated by sudden cardiac death as the presenting feature, requiring a molecular autopsy for diagnosis, not just a standard test on autopsy. If untreated, CPVT has a high mortality rate, reaching 30-35% by 30 years of age [4]. 

In a study conducted by Hayashi et al., the prescription of beta blockers lowered the event rate but did not prevent arrhythmia [3]. Implantable cardioverter defibrillator (ICD) may not terminate CPVT and is further associated with long-term complications that may be more prevalent in patients who have cardiac implantable devices placed early in life [5]. A study by Roston et al. compared the efficacy of current treatment modalities. Although some therapies have shown promising results, with beta blockers being considered the gold standard of care, none conclusively proves the prevention of arrhythmia, or reliably treats it. The current standard of care encompasses beta-blocker use to minimize adrenergic input, calcium channel blockers, flecainide, ICD implantation, and left cardiac sympathetic denervation [6].

The development of gene therapy provides hope for patients to minimize medical interventions as well as the risk of sudden death. It is broadly defined as a mode of therapy based on the introduction of genetically modified genes into cells by viral vectors, oligonucleotides, and/or modified mRNA. These novel therapies allow the restoration of adequate functional proteins, which can prevent the onset of arrhythmias. This may ameliorate disease symptoms and pave the way for the delivery of a permanent cure, improving both quality and quantity of life. In this narrative review, we will examine in detail the epidemiology, pathophysiology, risk factors, clinical features, diagnosis, and differential diagnosis, the genetic mutations involved, and the current standard of care for CPVT, along with a discussion of current gene therapy research and the potential for treatment in human patients.

## Review

Epidemiology and genetic associations

In a study conducted by Pérez-Riera et al., CPVT has an estimated incidence of 1 in 5000/10000. The initial presentation ranges from two years to 21 years of age, although certain sporadic cases have even presented up to 40 years of age, with a poor prognosis ranging up to 50% mortality by 20 years of age [7].

CPVT has been classified by gene mutation into five subtypes: CPVT1 (involving the RyR2 gene) and CPVT3 (TRDN [triadin]) present with symptoms at around 10 years of age; CPVT2 (involving the CASQ2 gene) manifests symptoms at around seven years of age, while CPVT4 (involving the ankyrin-2 gene [ANK2]) and CPVT5 (involving the potassium inwardly-rectifying channel subfamily J member-2 gene [KCNJ2]) presents with symptoms at four and 2.5 years of age. Familial occurrence was shown in about 30% of cases, and the inheritance pattern may be autosomal dominant (RyR2 gene) or autosomal recessive (CASQ2 gene). It has been seen more in women and genotype-negative patients. About 30% of patients with CPVT passed away before the age of 40 [7].

Mutations in the RyR2 gene are the most frequent form of CPVT, also known as CPVT1 (about 60% of cases) [8]. This gene translates to the cardiac ryanodine receptor, which is an intracellular Ca2+-release channel that is involved in cardiac excitation-contraction coupling. It is inherited in an autosomal dominant pattern with a male-to-female ratio of 1:1. Most commonly, it is due to a gain-of-function mutation in RyR2-encoded ryanodine receptor-2 [9].

The second variant of CPVT occurs due to mutations in the CASQ2 gene. CASQ2 encodes for the calsequestrin protein and has an incidence of 1-2%. It is most commonly an autosomal recessive (AR) trait with an M/F ratio of 1:1 [10].

Another variant, i.e., CPVT3, was found with the mutations in the TRDN gene, where 97 probands (index cases found to have phenotypical CPVT) were screened and three related variants were found, which encode for a protein that links RyR2 and CASQ2 in the sarcoplasmic reticulum (SR). Autosomal recessive inheritance phenotype with an M/F ratio of 1:1 is seen in <1% of patients with CPVT. It also has some overlap with the characteristic features of long QT syndrome. Finally, CPVT4-ANK2 mutations [11] and CPVT5-KCNJ2 mutations [12] were seen in a minority of CPVT patients (<1%), in an autosomal dominant manner with an M/F ratio of 1:1. In these variants, the calmodulin (CALM) protein is affected [13]. CALM, a calcium-binding protein, stabilizes RyR2 and decreases the opening of the RyR2 channel during diastole.

Pathophysiology

Cardiomyocytes use the excitation-contraction coupling (ECC) mechanism to convert action potentials into mechanical force when they are functioning normally. Sarcolemma depolarization in the ECC causes voltage-sensitive Ca2+ channels, located in the T-tubule, to open. This favours a current of calcium to move in, which then causes additional calcium release through the opening of the RyR receptors via a calcium-induced calcium release mechanism [14-18]. The RyR2 is a receptor that is based on the sarcoplasmic/endoplasmic reticulum membrane [17] and controls the large release of Ca2+ from intracellular stores through cardiac systole [18]. CPVT is a hereditary disease, due to a malfunction in Ca2+ ion transport or release from their storage in cardiac myocytes causing delay upon depolarization [14,16]. Several genes have been identified to be the culprit of a high percentage of patients affected by this disease [15]. These genes encode for proteins like RyR2, CASQ2, TRDN human, and calmodulin 1,2, and 3 (CALM1-3) that are in charge of controlling the release of calcium from the endoplasmic reticulum during excitation-contraction (EC) process [7].

**Figure 1:**
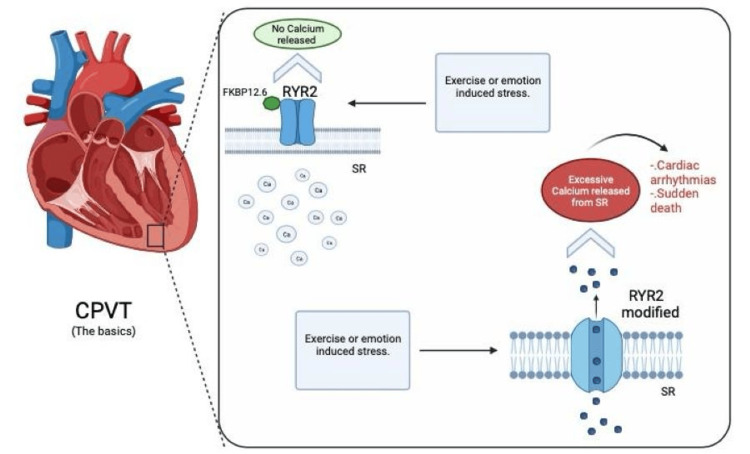
Ryanodine receptor-2 (RyR2) receptors under physiologic conditions release calcium from intracellular stores during the excitation-contraction process in cardiac muscles. Mutation of the RyR receptor causes structural changes in the protein, releasing an abnormal amount of calcium and producing arrhythmias in the presence of stimuli such as stress or emotions. SR: sarcoplasmic reticulum; RyR2: ryanodine receptor-2; CPVT: catecholaminergic polymorphic ventricular tachycardia Figure created by using BioRender.com

A relevant mechanism in the pathogenesis of PCVT involves the malfunction of RyR2 [17] as depicted in Figure 1. Several missense mutations of the gene were associated with two hereditary presentations of sudden death related to cardiac events: catecholaminergic polymorphic ventricular tachycardia and type 2 right ventricular cardiomyopathy with arrhythmogenic features [9]. Calstabin-2 (also known as FKBP12.6) is an important subunit that binds to the RyR2 complex, which helps to keep the channel closed, preventing the opening when the calcium signal is absent. The disconnection between calstabin-2 and RyR2 may cause structural changes in the channel protein that alter its sensitivity to calcium and increase the probability of opening, leading to abnormal calcium release and triggering arrhythmias [17]. CASQ2 is a protein with a significant function in regulating calcium levels within cardiac muscle cells and helps to maintain adequate calcium concentrations for physiologic muscle contraction and relaxation. Mutations in the CASQ2 interfere with its natural action, resulting in a buildup of calcium in the sarcoplasmic reticulum, which can trigger abnormal heart rhythms like CPVT [19].

Risk factors

The main clinical symptom that triggers suspicion of CPVT is syncope associated with exercise or emotion. These patients may have a family history of sudden death in children or syncope with similar characteristics [18]. Because the prognosis and clinical symptoms of this arrhythmia might be fatal, it is necessary to broaden the examination to uncover other CPVT instances within a family. Early diagnosis in childhood or adolescence is linked to a higher risk of future cardiac events [19,20]. Individuals with unstable ventricular arrhythmias, even if they are compliant with beta blocker medication, are also at high risk [20,21].

Clinical presentation and symptomatology

Most of the patients are asymptomatic throughout life but in the presence of a trigger event, such as emotional or physical stress, CPVT may present as syncope [22,23]. This is the most common symptom, followed by dyspnea, chest pain, dizziness, palpitations, and even cardiac arrest with a full recovery after the event or leading to sudden cardiac death in the setting of a normal structural heart. Sudden cardiac death may be the first symptom as well [4]. Even without a triggering stimulus, there have been reports of fatal cardiac episodes while performing routine everyday tasks [24]. Further challenging diagnosis, clinical examinations such as echocardiography, coronary angiography, and resting ECG are often normal [25].

Some patients can present with a seizure history during childhood or adolescence, more specifically in the first or second decade of life [23]. CPVT can manifest with distinct patterns of onset in both juveniles and adults. Hence, A bimodal distribution of the age of onset is reported, with a second onset peak in the third and fourth decades of life, suggesting a juvenile form as well as an adult form [26].

Disparity in age, ethnicity, and other sociodemographic factors

Males and adolescents may have a strong relationship with CPVT, possibly due to a higher chance of involvement in physically stressful activities such as sports, work, or other activities. Nevertheless, there are recent large cohort studies in 2017 that show no link between sex and the onset of symptoms and signs [27]. This was again supported by a more recent study in 2020 by Haugaa et al. who concluded that there is no conclusive proof that sex differences exist in illness penetrance, arrhythmic risk, or clinical presentation in CPVT. When those with cardiac symptoms presented, ethnicity was not related to the age of symptom onset [25].

Diagnosis and evaluation

The presence of clinical features like exercise or emotion-induced syncope [22,23], repeated syncopal attacks [28], and sometimes even the presentation of life-threatening arrhythmias should elicit a suspicion for CPVT [3,6]. Catecholamine surge-induced polymorphic ventricular tachycardia or bidirectional ventricular tachycardia points towards CPVT when accompanied by a heart that is structurally devoid of any defects and a normal resting electrocardiogram. Alternatively, CPVT can be diagnosed by conducting genetic testing or by identifying heterozygous disease-causing mutations in RyR2, CALM1-3, KCNJ2, or bi-allelic pathogenic variants in CASQ2, trans-2,3-enoyl-CoA reductase like (TECRL); or TRDN is pathognomonic [29-31]. Bidirectional VT, often known as the alternate 180°-QRS axis, is frequently the distinctive presentation of CPVT arrhythmias [28]. "Stable" QRS vector alternans may not always be present in people with CPVT, since some may also exhibit irregular polymorphic VT [32,33]. Despite the possibility of conditions like Andersen-Tawil syndrome and overdose of digoxin, the detection of bidirectional ventricular tachycardia is nearly pathognomonic for CPVT [20,34,35].

For diagnosis and evaluation of CPVT, the standard exercise stress test using the Bruce protocol or an equivalent protocol may be used. However, despite having unprovoked arrhythmias, studies show that sudden cardiac death is still possible in those with abnormal results during the protocol [33]. A study involving relatives of CPVT probands with no symptoms and with a mutation in RyR2 and CASQ2 indicated a high specificity of 97% and modest sensitivity of 50% for the exercise stress test in the diagnosis of CPVT [36-38]. These outcomes are in line with those reported by Bauce et al. [39] who, despite including symptomatic participants from families with pathogenic RYR2 mutations, found sensitivity and specificity to be 60 and 100%, respectively. In this context, a case study that used a modified "sprint" EST protocol to test its ability to produce positive results in cases that the Bruce protocol had ruled out is noteworthy [40]. This suggests that sprint EST may be more effective at inducing arrhythmias than the traditional Bruce protocol.

Depending on the phenotype, molecular gene testing methods can combine targeted methods like a multigene panel and comprehensive genomic testing. The multigene panel is most likely to show the genes with known involvement, and the comprehensive genomic studies comprise exome sequencing, which has the advantage of including genes that have recently been identified as causing CPVT. Genome sequencing is also possible [41]. Recent evidence suggested a possible candidate gene, agrin (AGRN), for the catecholaminergic polymorphic ventricular tachycardia, though functional studies are necessary to fully understand their implications and the pathophysiology in CPVT [42,43]. The exercise-stress test is quicker and less expensive than genetic testing to confirm results, but a study revealed a false-positive result of 6% and a false-negative result of 32%. Hence, genetic screening is imperative even in symptom-free relatives of confirmed CPVT probands to avoid unnecessary treatment or restriction in those who do not carry the mutation, along with identifying and preventing possible arrhythmias in those who do carry the mutation [37].

The possibility of negative results with genetic testing for arrhythmic genes has resulted in employing sustained monitoring with implanted loop monitors or an epinephrine challenge to diagnose CPVT [4]. However, provocation testing by injecting epinephrine or isoproterenol intravenously can also be used to identify people who are unable to exercise but have concealed CPVT [44]. A borderline test consists of non-sustained monomorphic ventricular tachycardia, paroxysmal ventricular contractions or polymorphic couplets [45]; a positive test results in more than two beats of polymorphic VT or incidence of bi-directional ventricular tachycardia.

Positive clinical signs and a poor prognosis call for the screening of relatives of a confirmed proband. Both first and second-degree relatives undergo screening. A CPVT phenotype is present in around 50% of the relatives who were found to carry the RYR2 mutation by cascade screening. Therefore, when a pathogenic gene is found in the proband, genetic testing of the family is advised, not only to facilitate the diagnosis of silent carriers but also to enable the use of preventative medication and conduct an analysis of the reproductive risk [2,46].

Differential diagnosis

Short-coupled ventricular tachycardia (SC-torsade de pointes [TdP]) might present with polymorphic ventricular arrhythmias similar to that of CPVT. Though it presents that way in an anatomically unremarkable heart and a normal baseline electrocardiogram, SC-TdP has a distinction of not being associated with adrenergic stimuli and is not accompanied by bi-directional patterned arrhythmias, unlike CPVT. It is critical to distinguish between the two illnesses since SC-TdP has no known effective treatment, but CPVT typically responds to flecainide and beta-blockers [47].

Young adults with arrhythmogenic right ventricular cardiomyopathy (ARVC) are more susceptible to ventricular tachycardia and sudden cardiac death. However, this condition often manifests as monomorphic ventricular tachycardia, an abnormal ECG, and structural abnormalities on cardiac imaging [48]. This plakophilin-2 (PKP2) dependent electropathy progresses regardless of structural disease and can also present with exercise-induced symptoms before overt cardiomyopathy develops. This clinical overlap was elucidated in one study where some patients with a clinical diagnosis of CPVT exhibited PKP2 pathogenic mutation [49], emphasizing the need for rigorous ongoing structural evaluation with echocardiogram or cardiac magnetic resonance imaging.

Exercise-related syncope can be a symptom of LQTS, usually in the LQT1 variant [50]. Andersen Tawil syndrome, a variant of long QT syndrome, can also show bidirectional ventricular tachycardia. However, this condition can be distinguished from CPVT by prolonged QT interval on the ECG, clinical features related to the syndrome, low risk of sudden death, and the lack of uniform association with adrenergic activity [33].

Genetic mutations in CPVT

The vital role of the sarcoplasmic reticulum in the functionality of the heart is carried out in part by the ryanodine receptors (RyRs). These are sizable calcium channels located in the sarcoplasmic reticulum and release calcium from the intracellular reservoir as a part of the excitation-contraction coupling mechanism. Research conducted in 1999 by Swan et al. identified a novel, highly malignant cardiac condition resulting in exercise-induced polymorphic ventricular tachycardia in anatomically healthy hearts [32]. It linked the mutation to chromosome 1q42-q43 [33]. They noted that this RyR2 gene mutation was present in conjunction with clinically competent myocardium. This combination was predominantly noted in the autosomal dominant variant of CPVT [51].

A missense mutation in a highly conserved region of the CASQ2 gene has also been connected with phenotypical CPVT; this mutation was seen in the autosomal recessive type [51]. The CASQ2 protein serves as the primary Ca2+ reservoir in the sarcoplasmic reticulum (SR) of cardiac myocytes and is part of a protein complex that also contains the ryanodine receptor [51]. Its primary job is to assist in controlling calcium release. The basis of diagnosis for this disorder significantly relies on genetic testing because there are no anatomical defects. In a study completed under the guidance of Bauce et al. [39], the importance of genetic testing for asymptomatic carriers was emphasized.

Twenty-three exons of RyR2 that were previously linked to CPVT1, as well as an extensive examination of all exons translated in CASQ2 (CPVT2), KCNQ1 (LQT1), KCNH2 (LQT2), SCN5A (LQT3), KCNE1 (LQT5), KCNE2 (LQT6), and KCNJ2 (Andersen-Tawil syndrome [ATS1] or LQT7), were analyzed for mutations. In the final analysis, four females have been determined to have known ATS1 or LQT5-associated mutations, while CPVT-1 variants have been identified in P164S, V186M, S3938R, and T4196A. However, more than 400 reference alleles [52] lacked these changes.

If genetic testing reveals insufficient evidence of a ryanodine receptor mutation, but the clinical picture is still consistent with the finding of CPVT, suspicion of Andersen-Tawil syndrome and long QT syndrome should be explored [52].

Standard treatment

Pharmacological Treatment

The current approach to the management of CPVT involves a combination of multiple strategies that can complement each other [29]. The main goal of treatment is to decrease the incidence of arrhythmias. This can be achieved by combining different approaches such as lifestyle changes, pharmacological treatments, implantable cardiac defibrillators, or left cardiac sympathetic denervation. Guidelines advise staying away from stressful situations, challenging activities, and competitive sports because these events cause a release of catecholamines, which in turn triggers the CPVT [29].

Beta-blockers are the cornerstone of therapy and should be titrated to the maximum dose that is tolerated by the patient [29]. Some clinical evidence points to nadolol's superiority, at the dose of 1-2 mg per kg per day [3,53]. Since occurrences of cardiac arrest have been observed, asymptomatic carriers of harmful gene mutations should also be provided with beta-blockers [29,37]. Beta-blocker medication does not, however, offer total protection [23].

In those patients who continue to have arrhythmic events despite beta-blockade, flecainide add-on therapy should be considered at 2-3 mg per kg daily [29]. Flecainide prevents the RyR2-driven calcium release in CASQ2 knockout mice and prevents arrhythmic events, as demonstrated by Watanabe et al. [54]. However, later research called into doubt the underlying process for the impact of flecainide in CPVT [55]. It offered some evidence that this efficacy is mediated by a decrease in the availability of sodium channels and a resultant increase in arrhythmogenic threshold [55]. This theory was later supported by other research; however, flecainide has not been shown to have long-term efficacy in preventing potentially fatal arrhythmias [56].

Novel pharmacological agents: As prospective innovative treatments for CPVT, many therapeutic approaches that aim to restore damaged pathways that encourage arrhythmogenesis have been proposed. Several studies have reported that K201, a 1,4-benzothiazepine derivative, stabilised calstabin2-RyR2 binding and prevented arrhythmias, likely due to a structural resemblance to diltiazem [57,58]. Similarly, Yano and colleagues found that giving dantrolene to mice with the p.Arg2472Ser heterozygous mutation prevented arrhythmias. Dantrolene changes how certain domains of RyR2 interact and increases the probability of the channel being open. Researchers additionally demonstrated that dantrolene repairs the connection between these domains, therefore preventing ventricular arrhythmias [59,60]. Furthermore, a study demonstrated that ent-(+)-verticilide, an artificial derivative of the insecticide verticilide, reduces delayed after-depolarisation by selectively inhibiting RyR2-led calcium release in murine cardiac cells affected by CASQ2 gene mutation. This arrhythmia-preventing mechanism is not clearly established but seems to be independent of RyR2 phosphorylation or binding of RyR2 to accessory proteins. In contrast, dantrolene and K201 influence two out of three previously known mechanisms of RyR2 malfunction. Currently, dantrolene was found to reduce arrhythmia in patients with defective RyR2 domains, while clinical studies have not been conducted on K201 and ent-(+)-verticilide [61].

Non-pharmacological Treatment

Cardiac sympathetic denervation (CSD) (left-sided) can be an option for individuals who experience symptoms while receiving the optimal medical therapy or who are unable to tolerate BB treatment (Class IIb recommendation) [29]. However, long-term anti-arrhythmic protection is not assured by this [62]. Those who sustain a cardiac arrest or those who have syncope or sustained ventricular tachycardia despite receiving appropriate treatment may be considered for an implanted cardioverter-defibrillator (ICD) (Class Ic recommendation) [29]. Compliance with proper BB therapy, meticulous implantable device programming, sufficient time gap before shock administration, and high cut-off heart rates for detection are required [63] to prevent unnecessary shocks that might have a proarrhythmic effect. ICDs have been documented to fail in correcting the rhythm for CPVT patients, and CPVT-2 individuals appear to experience this failure more commonly [64].

One of the most effective and cutting-edge procedures is minimally invasive bilateral heart sympathetic denervation. Both single-lung ventilation and two-lung ventilation can be used during the process. Generally, the patient is extubated the same day as the procedure and released three days later. One-sided xerosis of the hand, differences in temperature or skin colour between the left and right sides of the face, diaphoresis, postoperative pain over the shoulder blades, drooping of the eyelids, decreased pupil size, and chest pain are potential adverse effects in their decreasing order [65]. CSD is a popular therapy option for CPVT patients due to its low surgical risk, quick hospital stay, typically tolerable side effects, good arrhythmia control, and increased quality of life. As a result, it is advised that CSD be considered as an approach following the appropriate medical treatment and prior to implantation of implantable cardioverter-defibrillator [44]. Implantable device placement continues to be promoted by global guidelines; however, a large observational study involving ICD placement in undiagnosed cases of CPVT with a prior cardiac arrest and no medical treatment showed no difference in rates of sudden cardiac death from those who did not have an ICD placed. Instead, a significant prevalence of ICD shocks which did not improve survival chance, inappropriate ICD shocks, and several other problems related to the device were linked to the ICD [66].

Pharmacological and non-pharmacological treatment modalities are illustrated in Table 1.

**Table 1:** Treatment modalities for CPVT CPVT: catecholaminergic polymorphic ventricular tachycardia; SCD: sudden cardiac death

Treatment modality	Relevant studies	Merits of using the modality in CPVT treatment	Limitations
Beta-blockers	Priori et al. [29]	SCD incidence is noticeably decreased in people on beta-blockers	Does not confer complete protection
Flecainide	Watanabe et al. [54]; Bannister et al. [56]	Prevents life-threatening arrhythmias	Lacking long-term outcome data on the efficacy
Dantrolene	Penttinen et al. [61]	Reduction in the arrhythmic burden	Only patients with ryanodine receptor-2 mutations in certain domains exhibited antiarrhythmic effects; those with mutations in other domains exhibited almost no effect
Implantable cardioverter defibrillator	Priori et al. [29]; Van der Werf et al. [66]	Recommended by guidelines for individuals who sustain a cardiac arrest or those who have syncope or sustained ventricular tachycardia while receiving appropriate treatment	(a) Incidence of inappropriate or pro-arrhythmogenic shocks [66]; (b) no reduction in the occurrence of the SCD [66]
Cardiac sympathetic denervation	Bansal et al. [43]; Marai et al. [62]	Excellent arrhythmic control with improved quality of life [65]	Long-term complete antiarrhythmic protection cannot be ensured [62]

Lifestyle changes and agents to avoid

Competitive sports and other forms of rigorous activity are never advised for people with CPVT. All people who exhibit exercise-induced arrhythmias should refrain from physical activity. However, patients receiving treatment and exhibiting good arrhythmic control on that treatment may be allowed modest exercise. It is crucial to remember that efficacy must frequently be retested [67]. Those with mutated genes linked to CPVT but without a clinical picture of exercise-induced arrhythmia may be best served by the recommendation to avoid strenuous physical activity since the risk of arrhythmia during sports is unknown. Also, the drug digitalis should be avoided by individuals with CPVT due to prolonged after-depolarization [47].

However, some patients, especially those who are young, are unable to follow the exercise restriction. An insertable cardiac monitor (ICM) was successfully employed in a patient in a trial that was conducted in a circumstance similar to this one, where the ICM recorded the episodes and identified how much intense activity causes arrhythmia, which in turn allowed for effective management of the problem [68,69].

Surveillance

Patients with CPVT must be followed by a cardiologist at least yearly if not biannually, particularly up to puberty. Body weight is especially labile during this time, and dosage must be adjusted to body weight as much as possible. Various devices with the capability of monitoring the heart rate are increasingly available commercially and can be used for sports participation so that the intensity of physical activity can be optimized. However, they do not substitute regular medical follow-ups. Exercise stress testing allows for risk stratification and personalization of advice regarding exercise intensity [47].

Genetic Counseling

The parents of a proband should undergo molecular genetic testing if a proband has a confirmed molecular diagnosis. This will establish the heterozygosity for a potential CPVT-causing mutation and allow a reasonable risk assessment for recurrence. Probands with autosomal recessive mutations should have parental screening by a maximal exercise stress test. Clinical screening is advised in people who have a heterozygous pathogenic variation in CASQ2 as may exhibit a mild CPVT phenotype [47].

The pre-conception period is ideal to assess reproductive risk and discuss the considerations for prenatal genetic testing. Genetic counselling is a reasonable option for young, affected or at-risk individuals, including discussions of potential dangers to offspring and available reproductive alternatives. Prenatal testing of a fetus at increased risk and preimplantation genetic testing are viable options after the pathogenic mutations have been detected in an affected family member [47].

Potential gene therapy: the emergence of new studies on gene therapy (pre-clinical / clinical)

Pre-clinical research has ensured a better understanding of current mechanisms of treatment. However, due to restrictions on gene testing, there are gaps in clinical trials. One study by Kurtzwald-Josefson et al. utilized a murine model of CPVT2 to improve the understanding of the mechanism behind the recessive type of CPVT2 [70]. Twelve-week-old mice with this mutation were injected with adeno-associated virus (AAV) particles that expressed the CASQ2 gene (AAVCASQ2) to replenish the murine levels; after seven to eight weeks provocation trials were run under telemetry monitoring. This showed that the effectiveness of the antiarrhythmic action of AAVCASQ2 was dependent on in vivo CASQ2 levels (>33% of normal), providing a potential therapy for CPVT2 [70]. 

Hajjar et al.'s research [71] found that patients receiving high-dose SERCA2a gene therapy when compared to control for the treatment of chronic heart failure showed a significantly lower clinical event rate three years after the gene transfer. This was in accordance with the successful conclusion of phases 1 and 2 of this study [72,73]. They also demonstrated that patients who underwent high-dose SERCA2a therapy resulted in persistence of the SERCA2a gene in cardiac tissues for up to 31 months after transfer. These preliminary results indicate this method is safe and not connected with any virus-related adverse events, despite a relatively small sample size and short follow-up period. This research opens avenues towards cardiac gene therapy becoming a potentially useful therapy for the treatment of hereditary cardiac disorders [71].

Potential gene therapies

Due to the limitations in the current treatment options, gene therapy has garnered significant attention in recent times for the treatment of CPVT. Gene therapy refers to a therapeutic approach that involves introducing genetic material into cells, which can be accomplished using various techniques such as viral vectors, oligonucleotides, and modified mRNA [74]. This approach aims to address genetic defects by either repairing or replacing faulty genes, regulating gene expression, or producing therapeutic proteins.

Recently gene therapy has yielded promising results in inherited cardiac arrhythmias. An experiment involving the use of the AAT9 vector to transfer the cDNA of cardiac calsequestrin in mice along with cytomegalovirus promoter and GFP as a reporter gene in a murine model (2011) is worth mentioning. Murine homozygous CASQ2 knockout models were infected with AAVCASQ2 via intraperitoneal injection immediately after birth and clinically evaluated at 20 weeks of age. In a range of in vitro and in vivo experiments that followed, researchers found that the gene transfer was successful in correcting all abnormalities attributed to the knockout gene. In the following experiments, the efficacy of gene replacement therapy in these R33Q knockout mice was studied. When mice were infected at birth with gene therapy, it prevented all the clinical manifestations of CPVT. Similarly, mice infected as adults showed complete regression of disease manifestations, including life-threatening ventricular arrhythmias. Remarkably, even after 12 months of being given a single dose of gene therapy, the mice continued to exhibit full therapeutic effect [75]. These AAVCASQ2 experiments are slowly paving a road towards a long-lasting therapy with the potential to ameliorate and even cure CPVT2 [76].

Another study by Lodola et al. involved the creation of cardiac myocytes (CMs) from induced pluripotent stem cells (iPSCs) from a patient known to have homozygous CASQ2-G112+5X mutation. The viral vector AAV9 carried a normal variant of the human CASQ2 gene in its natural state (AAV9-hCASQ2) and infected the CMs in vitro. The researchers were able to resolve the functional deficiencies (percentage of delayed after depolarizations, calcium spark density, and duration, etc) found in the patient-specific iPSC-derived CMs by inserting the healthy CASQ2 gene and restoring the normal production of calsequestrin-2 protein. The success of AAV9-mediated gene therapy for human CMs with known CPVT2 in vitro supports the theory that gene therapy may prove to be curative with further study in human variants of CPVT [77].

## Conclusions

The pathophysiological basis for dysfunctional calcium regulation in CPVT has been thoroughly documented. The limitation of current treatment options have contributed towards the attention given to cardiac gene therapy in the last 10 years since the first study in a mouse knock-out model was suggested. Adeno-associated viral vectors in cardiac gene therapies have been successfully started in human trials for diseases like heart failure and cardiovascular disease but for CVPT, this treatment has only been tested on murine models. The biggest limitations have been its rare population and the need for genetic testing to confirm diagnosis to be included in human trials as well as a lack of funding since the market for a clinically applicable gene therapy for CPVT is considered small in comparison to the amount of funds needed for its development. Nevertheless, great advances have been made in the last decade that give rise to hope for the future, where these novel therapies provide adequate protein function and prevent medical interventions or even fatal outcomes such as sudden cardiac death for patients with inherited channelopathies.
